# Diseases of the Pancreas

**Published:** 1896-01-04

**Authors:** 


### DISEASES OF THE PANCREAS.

**Pancreatic Cysts and Rupture of the Pancreas.**—Leith[1] writes a more or less extensive paper upon ruptures of the pancreas, and the connection of that injury with pancreatic cysts. He refers to the protected site of the pancreas, and mentions that recorded cases show that rupture of that gland very rarely occurs without serious injury to other viscera, the liver, spleen, duodenum, kidney, and, in one case, the lung being torn. Dr. Leith records two fresh cases of rupture of the pancreas, one of which came under his own notice; the notes of the other were given him by Dr. Goldman, of Freiburg, in Baden. In these also the pancreas had not suffered alone; in one the third part of the duodenum, and in the other the spleen, was ruptured. In Leith's case, a boy aged four years was kicked in the epigastrium by a horse; in the other, that of Goldman, a young man received a heavy blow from a falling packing case, which struck his abdomen. The boy lived only ten hours after reeeiving the blow, but the young man apparently longer; the time of death, however, is not stated. The blood effused in Dr. Leith's case amounted to about three ounces, and was situated around the pancreas behind the peritoneum. The hæmorrhage in the case of Dr. Goldman was more extensive, and was situated, apparently, in the lesser peritoneal cavity. After briefly reviewing the previously recorded cases of rupture of the pancreas, seven in number, Leith refers to those cases of so-called pancreatic cysts that have been supposed to arise from injury to the gland, and reviews the theories suggested by Kuhlenkampff and Senn to account for their formation. He seems disposed to accept the view of Senn, that the cyst may originate in a hæmorrhage, and quotes Cathcart as having further elaborated it. Cathcart supposed that injury causes a laceration of the gland, which laceration is followed by extravasation of blood. Not only blood, however, but pancreatic secretion escapes from torn ducts, and this secretion will be continually added to the hæmatoma. The collection of fluid thus produced probably becomes irritating in character, and excites the formation of a capsule around it. Leith mentions Jordon Lloyd as believing in this mode of origin of pancreatic cysts, and Theodore Fisher, as thinking that the cysts need not originate in hæmorrhages into the substance of the pancreas, but in a blood effusion in the neighbourhood of the gland. Dr. Leith proceeds later to quote several of the cases referred to by Fisher, to show that extensive hæmorrhages and hæmorrhagic cysts unconnected with the pancreas may occur in the layers of the peritoneum, and refers to the point upon which that writer lays stress, that it may be difficult to distinguish a cyst in the peritoneum from one associated with the pancreas. Since we are not justified in supposing that all cysts in the epigastrium unconnected with the liver or spleen are pancreatic in origin, Leith suggests that the term epigastric should be applied to them, unless examination of the contained fluid gives definite evidence of connection with the pancreas. At the end of his paper Dr. Leith refers to the treatment of these cysts. He mentions that aspiration is frequently ineffectual and sometimes dangerous, and that extirpation may prove fatal. Experience shows laparotomy with drainage to be by far the most successful method. He quotes Krecke as saying that of twenty-seven cases treated by section and drainage all recovered, but that of six cases treated by dissecting out the cyst three died. Cathcart and Littlewood are referred to as speaking of the ease with which a counter-opening can be made posteriorly in these cases in order to obtain better drainage, and it occurred to Dr. Leith that the cyst might be attacked primarily from behind. Experiments on the cadaver showed that from a point just below the twelfth rib, about three inches from the spine, the tail of the pancreas was easily reached. By altering the obliquity of the exploring probe the lesser peritoneal could be entered by going above the pancreas, or the general peritoneal cavity by directing the instrument below that gland and the transverse mesocolon. Dr. Leith further mentions that Mr. Cotterell adopted this method with success in a case of pancreatic cyst under his care in the Edinburgh Royal Infirmary.

**Acute Pancreatitis, Parapancreatic Abscess and Fat Necrosis.**—W. S. Thayer[2] records a case of pancreatitis with associated abdominal abscess, the main interest of which lies in the fact that the abscess was opened and drained and the patient recovered. It occurred in a man aged thirty-four, who during a year and a half suffered from severe attacks of cramp-like pain in the region of the epigastrium and umbilicus. During the fifth attack a swelling was noticed just above the umbilicus. There was fever, and he became delirious. He was admitted into the Johns Hopkins Hospital, under the care of Dr. Halstead. The stomach was distended, and gave a markedly tympanitic note on percussion, but below it and just above the umbilicus, there was a deep ill-defined mass. The fever continued, and the mass was suspected to be a collection of pus, the situation of which suggested the pancreas as its probable starting point. An exploratory operation was performed, and a mass of adherent fatty tissue found, filled with small white nodules of fat necrosis. Nothing more was done at this operation. Ten days later a further examination was made, when a cavity containing thick brownish-yellow pus, with large masses and shreds of necrotic fat, was discovered. The cavity was drained, and three months later the patient left the hospital quite well. Dr. Thayer briefly refers to the varieties of pancreatitis, but mainly directs his attention to the fat necrosis, which he considers the most interesting feature of the case. He mentions the theories advanced to account for its existence, and inclines to the view of Langerhans, that the necrosis follows a primary lesion of the pancreas, which allows the escape of a fat-splitting ferment from the gland into the surrounding tissue. This view of the production of fat necrosis can, perhaps, hardly be said to be supported by two cases of the same disease recorded by Stockton and Williams,[3] since in neither was the pancreas markedly affected. Both cases occurred in Swedes, the first aged fifty-four, the second thirty-nine. The fat of the peritoneum in both instances was dotted over with the small white nodules characteristic of fat necrosis. In one the pancreas lay in a cavity containing cheesy material resembling pus, and in the other the gland presented a few white spots of necrosis. Microscopic examination, however, of both these glands is said to have shown no changes that the writers had not seen in the perfectly healthy pancreas. Clinically, the onset of symptoms in each case was more gradual than in cases in which the pancreas has been seriously affected. The older man was taken ill in Liverpool with what was thought to be subacute peritonitis. In three days, however, he felt sufficiently well to start for New York, but on the voyage became much worse, and the ship's doctor was surprised that he arrived alive. When seen in New York there was great prostration, abdominal pain and tenderness, a frequent feeble pulse, and slightly raised temperature. The fæces were natural. In spite of his condition he travelled to his home in Buffalo, but died a few hours after his arrival. The other patient had been suffering from abdominal pain and nausea for a few weeks before he came under observation. At that time he was anæmic, and his skin had an icteric tinge. There was great prostration, sharp lancenating abdominal pain and abdominal tenderness. Constipation and occasional vomiting were present. The pulse was feeble, 80 to 90 to the minute. Death occurred the following day, and the post-mortem examination showed the fat necrosis of the peritoneum already mentioned.

[1] Lancet, Sept. 28th, 1895; Edinburgh Med. Journal, Nov., 1895. [2] American Journal of the Medical Sciences, Oct., 1895. [3] Ibid Sept. 1895.

